# The Balance Metabolism Safety Net: Integration of Stress Signals by Interacting Transcriptional Factors in *Streptomyces* and Related Actinobacteria

**DOI:** 10.3389/fmicb.2019.03120

**Published:** 2020-01-22

**Authors:** Juan F. Martín, Paloma Liras

**Affiliations:** Área de Microbiología, Departamento de Biología Molecular, Universidad de León, León, Spain

**Keywords:** Actinobacteria, stress signals, overlapping binding sequences, transcriptional factors, sigma factors, cyclic nucleotides, safety net, signal integration nodes

## Abstract

Soil dwelling *Streptomyces* species are faced with large variations in carbon or nitrogen sources, phosphate, oxygen, iron, sulfur, and other nutrients. These drastic changes in key nutrients result in an unbalanced metabolism that have undesirable consequences for growth, cell differentiation, reproduction, and secondary metabolites biosynthesis. In the last decades evidence has accumulated indicating that mechanisms to correct metabolic unbalances in *Streptomyces* species take place at the transcriptional level, mediated by different transcriptional factors. For example, the master regulator PhoP and the large SARP-type regulator AfsR bind to overlapping sequences in the *afsS* promoter and, therefore, compete in the integration of signals of phosphate starvation and *S*-adenosylmethionine (SAM) concentrations. The cross-talk between phosphate control of metabolism, mediated by the PhoR–PhoP system, and the pleiotropic orphan nitrogen regulator GlnR, is very interesting; PhoP represses GlnR and other nitrogen metabolism genes. The mechanisms of control by GlnR of several promoters of ATP binding cassettes (ABC) sugar transporters and carbon metabolism are highly elaborated. Another important cross-talk that governs nitrogen metabolism involves the competition between GlnR and the transcriptional factor MtrA. GlnR and MtrA exert opposite effects on expression of nitrogen metabolism genes. MtrA, under nitrogen rich conditions, represses expression of nitrogen assimilation and regulatory genes, including GlnR, and competes with GlnR for the GlnR binding sites. Strikingly, these sites also bind to PhoP. Novel examples of interacting transcriptional factors, discovered recently, are discussed to provide a broad view of this interactions. Altogether, these findings indicate that cross-talks between the major transcriptional factors protect the cell metabolic balance. A detailed analysis of the transcriptional factors binding sequences suggests that the transcriptional factors interact with specific regions, either by overlapping the recognition sequence of other factors or by binding to adjacent sites in those regions. Additional interactions on the regulatory backbone are provided by sigma factors, highly phosphorylated nucleotides, cyclic dinucleotides, and small ligands that interact with cognate receptor proteins and with TetR-type transcriptional regulators. We propose to define the signal integration DNA regions (so called integrator sites) that assemble responses to different stress, nutritional or environmental signals. These integrator sites constitute nodes recognized by two, three, or more transcriptional factors to compensate the unbalances produced by metabolic stresses. This interplay mechanism acts as a safety net to prevent major damage to the metabolism under extreme nutritional and environmental conditions.

## Coordination of Primary and Secondary Metabolism in *Streptomyces:* Interaction of Multiple Transcriptional Factors at Signal Integration Sites

**FIGURE 1:**
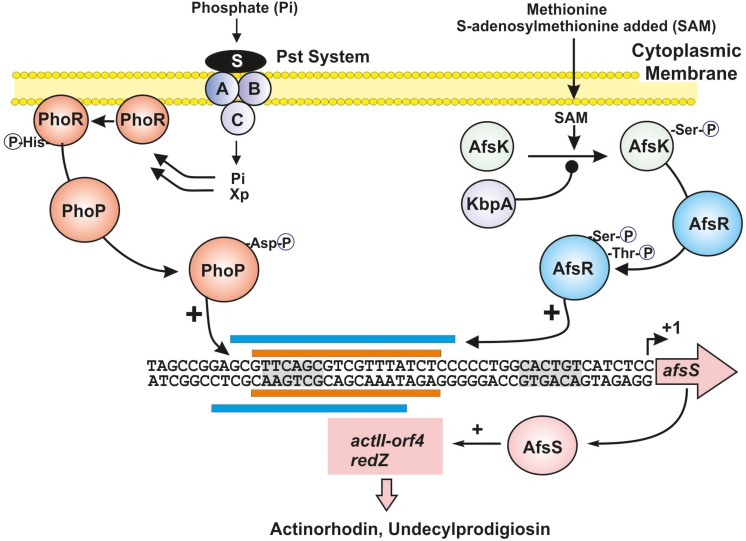
Integration of phosphate limitation and *S*-adenosylmethionine signals through overlapping transcriptional factors. The TCS regulatory proteins PhoR and PhoP are shown in dark orange. Xp indicates the proposed intracellular signal in *B. subtilis.* The methionine signal transduction cascade through AfsK (green sphere) and AfsR (blue sphere) is shown at the right site; KbpA (purple sphere) acts as an inhibitor of AfsK phosphorylation. The sites for PhoP and AfsR binding, in the region upstream of the *afsS* gene, are shown with orange and blue bars, respectively. The -10 and -35 sites (gray shadows) and the transcription start point of the *afsS* gene are indicated. Positive regulation is indicated by arrows and negative regulations by black spheres. See text for additional details.

Evidence accumulated in the last decades shows that coordination of primary and secondary metabolism takes place by multiple interactions of pleiotropic and cluster situated transcriptional factors that work in networks and cascades (Martín and Liras, 2010; van Wezel and McDowall, 2011; Martín et al., 2012b; Liu et al., 2013; Romero-Rodríguez et al., 2016a, b). Some pleiotropic transcriptional factors, such as PhoP, GlnR, or MtrA, control, directly or indirectly, hundreds of reactions in the bacterial cells, and we will refer to them in this article as master regulators. Master transcriptional factors interact with upstream regions of cluster situated regulatory genes. This is the case of several transcriptional factors that regulate the *act* and *red* clusters in *Streptomyces coelicolor.* The promoter region of the actinorhodin regulatory gene *actII-orf4* is recognized by at least nine transcriptional factors of different families, including GlnR, AfsS, AfsQ1, AdpA, AtrA, DasR, DraR, AbsA2, and AbsC (Floriano and Bibb, 1996; Ohnishi et al., 2005; Uguru et al., 2005; McKenzie and Nodwell, 2007; Rigali et al., 2008; Yu et al., 2012; He J.M. et al., 2016; Lewis et al., 2019). This phenomenon raises the question of how these transcriptional factors compete for binding to the *actII-orf4* promoter region. In other words, these regions (hereafter named integrators sites) serve to integrate multiple signal cascades that respond to different nutritional and environmental stress signals in *Streptomyces* (Figure 1).

An important question is whether these different transcriptional factors interact in some way in the control of expression of a particular gene. Taking into account the large size of some transcriptional regulators (see below), it is likely that each of these transcriptional factors covers at least the major groove or a full turn of DNA (11 nucleotides). Moreover, some of these regulators, e.g., GlnR or PhoP, act as dimers or even oligomers and therefore cover a relatively large stretch of DNA. Mutations affecting these integrator sites alter not only the binding to one transcriptional factor but also to other interacting factors.

The interactions between transcriptional factors and regions upstream of some gene clusters are really elaborated and suggests that there is a fine tuning of the expression of important gene clusters by alternative transcriptional factors. There are many reports of putatively interacting transcriptional factors that affect the biosynthesis of secondary metabolites but the molecular evidence supporting those interactions is scarce. In this article we focus on the most relevant and best-known cases of overlapping interactions between transcriptional factors that allow us to get an insight into how the cells integrate inputs from environmental and nutritional stresses. Those studied for which there are experimental evidence of DNA binding and/or footprinting data are summarized in Table 1.

**TABLE 1:** Well-known examples of interacting transcriptional factors in Actinobacteria^1^.

Actinobacteria	Integrative DNA region	Interacting transcriptional factors	Input signals
*S. coelicolor*	*actII-orf4*	AdpA, AtrA, DasR, DraR, AbsA2, AfsQ1, AfsS	GBL, *N*-acetylglucosamine levels, high glutamate levels, Pi limitation, SAM levels
	*redZ*	DasR, AbsA2, AfsQ1	*N*-acetylglucosamine levels, high glutamate levels
	*afsS* promoter	PhoP, AfsR	Pi limitation, SAM level
	*phoRP* promoter	PhoP, AfsR	Pi limitation, SAM level
	*glnR* promoter	PhoP, AfsR, AfsQ1	Pi limitation, SAM level, high glutamate levels
	*pstS* promoter	PhoP, AfsR, AfsQ1	Pi limitation, SAM level, high glutamate levels
	*cdaR* promoter	PhoP, AfsQ1, AbsA2	Pi limitation, high glutamate levels
	*scbA* promoter	PhoP, ScbR	Pi limitation, GBL
	*glnA* promoter	PhoP, GlnR, MtrA	Pi limitation, nitrogen limitation, complex nitrogen source
	*amtB* promoter, a3b3 site	PhoP, GlnR, MtrA, AfsQ1	Pi limitation, nitrogen limitation, complex nitrogen source, high glutamate levels
	*glnII*	PhoP, GlnR, MtrA	Pi limitation, nitrogen limitation, complex nitrogen source
	*ureA*	GlnR, PhoP, MtrA	
	*glnR* promoter	PhoP, MtrA	Pi limitation, complex nitrogen source
	*nirB1B2C/D* promoter	GlnR, MtrA	Nitrogen limitation, complex nitrogen source
	*malEFG* promoter	MalR, GlnR	Maltose, nitrogen limitation
*S. avermitilis*	*aveR* promoter	PhoP, GlnR	Pi limitation, nitrogen limitation
	*olmR* promoter	GlnR, AveR	Nitrogen limitation
*S. roseosporus*	*atrA promoter*	PhoP, BldA	Pi limitation, GBL
*Sac. erythraea*	*pstS* promoter	PhoP, GlnR	Pi limitation, nitrogen limitation
	*malEFG* promoter	MalR, GlnR, CRP-like^2^	Maltose, nitrogen limitation

*^1^Only examples of interaction confirmed by DNA-binding and/or footprinting assays are included. ^2^CRP has been proposed to bind to the *malEFG* promoter but there is no experimental confirmation of this binding. See text for references and details.*

## Mechanism of Control of Phosphate Metabolism: The PhoP Master Regulator

Phosphate is an essential nutrients of living beings. Phosphate starvation causes the slow-down of primary metabolism and triggers production of secondary metabolites in different microorganisms (Martín and Demain, 1980). Phosphate control of metabolism is mediated by the two-component system (TCS) PhoR-PhoP (Hutchings et al., 2004; Martín et al., 2012b) that controls not only primary metabolism but also the biosynthesis of secondary metabolites (e.g., actinorhodin and undecylprodigiosin) in *Streptomyces lividans* and *S. coelicolor* (Sola-Landa et al., 2003, 2005; Ghorbel et al., 2006). The PhoP/PhoR TCS has been also studied in *Streptomyces natalensis*, *Streptomyces tsukubaensis*, *Streptomyces avermitilis*, *Streptomyces hygroscopicus* var. *geldanus*, and orthologous sequences have been found in most Actinobacteria genomes. The genes encoding these TCS are well conserved in all *Streptomyces* (Martín et al., 2017, 2019; Ordóñez-Robles et al., 2017b; Martínez-Castro et al., 2018). The *Streptomyces* PhoR-PhoP system belongs to class IIIA of TCSs (Hutchings et al., 2004). PhoR is a protein sensor kinase with signal transducer activity (Cheung and Hendrickson, 2010) of 426 amino acids in *S. lividans* and *S. coelicolor*. It is membrane-anchored through a stretch of hydrophobic amino acids, and contains a cytoplasmic C-terminal region that putatively serves to interact with the phosphate starvation signal, although in some other TCS, the sensor kinase may have an extra-cytoplasmic signal sensor region (McKenzie and Nodwell, 2009). However, in *Streptomyces* species it is unclear if the phosphate limitation signal that interacts with PhoR is extracellular or if it is an internal signal molecule (Xp in Figure 1). In *Bacillus subtilis* it has been proposed that the signal molecule is an intracellular intermediate of teichoic acid biosynthesis. This intermediate is an inhibitor of the PhoR autokinase activity and is regulated by the phosphate concentration. Under phosphate limitation conditions the concentration of this intermediate decreases and, therefore, the autophosphorylation of PhoR and transfer of the phosphate group to PhoP are enhanced (Botella et al., 2014). PhoP is a member of the OmpR family of DNA-binding response regulators (223 amino acids). Under phosphate starvation conditions, the PhoR sensor protein kinase self phosphorylates, and then transfers its phosphate group to a receiver aspartate (D^49^) in PhoP, that causes a rearrangement of the protein structure resulting in an activation of the DNA-binding domain in the carboxy-terminal region of this transcriptional regulator. The phosphorylated form of PhoP binds specific sequences named PHO boxes and generally activates, but occasionally represses, the expression of genes of the Pho regulon. The *Streptomyce*s PHO boxes are formed by direct repeat units (DRus) of 11 nucleotides of which seven nucleotides (GTTCACC) are well conserved (Sola-Landa et al., 2005; Rodríguez-García et al., 2007; Allenby et al., 2012; Santos-Beneit, 2015). Two DRus are required for binding of PhoP to DNA (Sola-Landa et al., 2008) in agreement with the binding of dimer forms of PhoP (Blanco et al., 2002).

### The PHO Regulon: PhoP-Regulated Genes

The core Pho regulon is formed by more than 100 genes, of which about 50 are well characterized (Santos-Beneit, 2015; Martín et al., 2017). Using chromatin immunoprecipitation, Allenby et al. (2012) found PhoP binding sites in 150 loci in *S. coelicolor*, not all of them corresponding to promoter regions of characterized genes. Phosphate depletion results in PhoP-mediated induction of genes involved in scavenging, transport, and mobilization of phosphate, and in repression of genes for utilization of nitrogen sources. PhoP reduces expression of genes for aerobic respiration and activates nitrate respiration genes. This master regulator activates expression of genes for teichuronic acid formation and decreases that of genes for phosphate-rich teichoic acid biosynthesis. In *S. coelicolor* PhoP repressed several differentiation genes that affect development and indirectly antibiotic biosynthesis (Rodríguez-García et al., 2007; Allenby et al., 2012; Martín et al., 2017).

### Modification of PhoP Binding Affinity to Recognition Sequences by Acylation at Lysine Residues

The chemical structure of some regulatory proteins of the OmpR family, which includes PhoP, is known (Martínez-Hackert and Stock, 1997; Blanco et al., 2002, 2012; He X. et al., 2016). The DNA binding domain (DBD) of PhoP, and other OmpR-family regulators, includes a winged helix turn helix (HTH) structure in the carboxyl terminal region, that recognizes the PhoP binding sequence (PHO boxes) in the promoters of the target genes (Li et al., 2008). This DBD is highly positively charged and, therefore, has a strong affinity for the negative charged DNA backbone. It is known that several regulatory proteins are modified by acylation at the lysine rich region of their DBD (Li et al., 2008; Thao and Escalante-Semerena, 2011; Tiffert et al., 2011; Yao et al., 2014).

Particularly relevant for this review article is the acylation of residues in the lysine rich region of OmpR family regulators (Luo et al., 2004; Matsuzaki et al., 2005; Qin et al., 2016) that may alter the binding affinity to their recognition sequences (Banerjee et al., 2016). Recently, it was reported that the PhoP regulator of *Saccharopolyspora erythraea* is propionylated by an acyl transferase encoded by the *acuA* gene (Xu et al., 2018). Mass spectrometry analysis of the acylated PhoP revealed that the PhoP protein is propionylated at lysines K^198^ or K^203^. The AcuA enzyme in other organisms utilizes acetyl-CoA as acetyl donor, but in this particular case appears to use propionyl-CoA (You et al., 2014).

Mutation of K^198^ and K^203^ to arginine, glutamate, or glutamine provided evidence that whereas mutation to arginine conserves the polarity of the DNA binding region, the mutation to glutamine (or glutamate) changes the charge of this region thus weakening the binding of the mutated PhoP to the DNA backbone. These observations were supported by electrophoretic mobility-shift assay (EMSA) which showed that the mutation to glutamate removed the binding ability of PhoP and, therefore, altered the control of expression of the Pho regulon genes (Xu et al., 2018). In conclusion, highly propionylated forms of PhoP decrease the binding affinity of PhoP to the PHO boxes in the DNA, and therefore weaken control of the Pho regulon.

Since the 1970s, it is known that erythromycin is formed by condensation of 7-three carbon units provided by one propionyl-CoA as starter unit and six methylmalonyl-CoA elongation units (Cortés et al., 1990). Propionate or propanol addition to *Sac. erythraea* cultures enhances erythromycin production (Guo et al., 2016). Interestingly, propionate addition to erythromycin producing cultures also enhances the propionylated form of PhoP thus reducing the intensity of phosphate control (Xu et al., 2018). This is another interesting aspect of cross-talk between phosphate regulation and carbon source utilization, particularly propionate. It is unclear, however, if acylation of Omp-type response regulators, other than PhoP, in *Streptomyces* may also produce similar alterations of their regulatory effects. Indeed, the GlnR nitrogen regulator is modified both by phosphorylation and acetylation (see below).

### Modification of AfsR by Phosphorylation: Involvement in the Transduction of the *S*-Adenosylmethionine Concentration Signal

Expression of actinorhodin and undecylprodigiosin genes in *S. coelicolor* is controlled by the AfsR regulator that belongs to the large size SARP family (Horinouchi, 2003). AfsR (993 amino acids) contains a DBD in its N-terminal region and an internal ATP/GTP binding pocket in the middle of the protein. The AfsR regulatory effect is exerted on expression of the *afsS* gene, encoding a small protein (63 amino acids) that controls expression of the *act* and *red* gene transcriptional regulators (Floriano and Bibb, 1996; Lee et al., 2002) by binding upstream of these cluster specific regulatory genes. AfsR recognizes and binds the promoter of the *afsS* gene, and this binding is essential since no transcription of *afsS* occurs in mutants lacking AfsR. The synthesis of AfsS in turns activates the *act* and *red* transcriptional regulators, and therefore enhances the biosynthesis of actinorhodin, undecylprodigiosin, and the calcium-dependent antibiotic (CDA).

AfsR is phosphorylated at serine/threonine residues by the AfsK kinase (Hong et al., 1991; Lee et al., 2002). Phosphorylation of AfsR increases significantly the binding affinity of AfsR to the *afsS* promoter and therefore enhances actinorhodin, undecylprodigiosin, and CDA production. In addition, phosphorylation of AfsR modulates the ATPase activity of this regulator, that is required to rearrange the closed RNA polymerase–AfsR complex in the *afsS* promoter to an open transcriptionally active form. Genes orthologous to *afsK* and *afsR* occur in several *Streptomyces* species genomes suggesting that the mechanism of regulation through this phosphorylation cascade is important in these actinobacteria. Noteworthy, the autophosphorylating activity of AfsK is inhibited by KbpA, a protein encoded by a gene located upstream of the *afsK* gene in *S. coelicolor.* The KbpA protein binds to the unphosphorylated AfsK and inhibits its self-phosphorylation at serine/threonine residues. In AfsK–AfsR “*in vitro*” reaction mixtures KbpA inhibits the transmission of phosphate from AfsK to AfsR (Umeyama and Horinouchi, 2001; Figure 1). In summary, KbpA acts as a negative regulator in the AfsK–AfsR phosphorylation cascade, an important mechanism for the control of secondary metabolism and differentiation in some *Streptomyces* species, e.g., sporulation in *Streptomyces griseus.* Disruption of *kbpA* increases the phosphorylation cascade flux and results in an increase of actinorhodin production in *S. coelicolor.*

An important advance was the finding that the AfsK/AfsR proteins serve to integrate the signal of *S*-adenosylmethionine (SAM) concentration level. Methionine is one of the major methyl donors in microbial metabolism. This amino acid regulates positively or negatively the biosynthesis of many antibiotics, including actinorhodin (Kim et al., 2003), although the methionine signal transduction cascade has not been elucidated until recently. The methylation reactions are mediated by SAM, an activated form of methionine (Fontecave et al., 2004). The stimulatory effect of SAM is not observed in mutants blocked in the genes *afsR*, *afsS*, or *afsK*, or in the specific regulator gene *actII-orf4* (Jin et al., 2011). NMR studies of the serine/threonine kinase AfsK showed that SAM interacts with the carboxy-terminal moiety of this protein kinase and through this interaction increases the autophosphorylation of the AfsK protein in a dose-dependent mode (Jin et al., 2011). These authors propose that enhanced autophosphorylation of AfsK transmits the SAM signal leading to an increase in phosphorylation of AfsR, that in turns activates *afsS*, and therefore the production of actinorhodin and undecylprodigiosin (Figure 1).

### Interplay of PhoP With Other Regulatory Factors: Binding to Overlapping DNA Sequences

Although phosphate control of actinorhodin and undecylprodigiosin biosynthesis in *S. coelicolor* is mediated by PhoP, no direct binding of PhoP to the region upstream of the translation start triplet of genes of the *act* and *red* gene clusters was observed (Martín, 2004; Santos-Beneit et al., 2009b; Allenby et al., 2012).

Rather, the phosphate control of these antibiotics biosynthesis is exerted by interference of PhoP and AfsR by binding to the same sequence in the promoter region of the *asfS* gene. Both phosphorylated PhoP and AfsR are competing positive regulators that integrate signals derived from different inputs (Lee et al., 2002; Santos-Beneit et al., 2009b, 2011b; Figure 1). Although both PhoP and AfsR overlap completely there is a preference for one particular nucleotide in the sequence, different for PhoP or AfsR. Indeed, nucleotides in the PHO and AfsR boxes at positions 2, 3, 4, and 6 are invariant but the nucleotide at position 5 may be a T or a G. When there is a G at position 5 it promotes binding of PhoP, whereas a T at this position promotes binding of AfsR. AfsR and PhoP compete not only in binding the PHO box upstream of *afsS* but also AfsR binds to PHO boxes in the upstream regions of *pstSCAB*, *glnR*, and *phoRP* (Santos-Beneit et al., 2009b, 2011b).

Additional examples of overlapping recognition sequences, by PhoP and other transcriptional factors, have been reported in the last decades. In one example PhoP controls directly *cdaR* expression, the cluster situated regulator of the CDA in *S*. *coelicolor*. In addition to PhoP, other master transcriptional factors, including AbsA2 and AfsQ1, bind the upstream sequence of *cdaR* (Allenby et al., 2012). Other interesting example is the regulation of the *scbA* gene, encoding the γ-butyrolactone synthase (D’Alia et al., 2011) by both PhoP and ScbR regulators (Allenby et al., 2012). Both *scbA* and *scbR* are expressed from a divergent promoter and expression of *scbA* is regulated by ScbR, while PhoP binding to the divergent promoter represses *scbA* expression. Therefore, there is a close connection of the regulation by the γ-butyrolactone system and the PhoP-mediated phosphate control in *S. coelicolor* (Allenby et al., 2012).

### Cross Regulation of Phosphate and Carbon Sources

Cross-talk regulation between phosphate and carbon sources has been described in *Corynebacterium glutamicum* and also in a few *Streptomyces* species. Initial studies in *S. lividans* on carbon source control of phosphate transport through the high affinity PstSCAB transport system reveal that formation and release of the PstS protein takes place only in cultures with high concentration of fructose, galactose, or mannose but not with other carbon sources (Díaz et al., 2005). The PstS phosphate binding protein is partially released from the PstSCAB complex located in the cells envelopes and this release seems to be due to glycosylation of the PstS protein (Díaz et al., 2005; Wehmeier et al., 2009). The released PstS protein becomes not functional and unable to bind phosphate (Esteban et al., 2008). Using Northern analysis of the *pstSCAB* gene and Western blot assays of the PstS protein in *S. lividans* and *S. coelicolor*, Esteban et al. (2008) observed that the *pstS* gene expression takes place in media containing glucose or fructose as carbon sources but does not occur in complex medium. A stretch of DNA upstream of the *pstS* translation start triplet, including 8 degenerated repeats of a sequence of 12 nucleotides, is involved in negative control of the transcription from the *pstS* promoter by fructose. This stretch of DNA is adjacent to, but does not overlap with, the well-characterized PHO box recognized by PhoP, though both DNA regions affect expression of the *pstS* gene. However, further detailed analysis of PhoP and the putative role of the repeated 12 nucleotides sequence in regulation by carbon sources needs to be made. Similarly, in *C. glutamicum* the GlxR regulator, which is a cAMP-dependent transcriptional regulator, functionally similar to CRP in Enterobacteria, regulates phosphate transport by binding to a sequence upstream of the phosphate transporter gene *pstS* under phosphate limiting condition and activates its expression (Panhorst et al., 2011).

Another interesting example in *S. coelicolor* is the regulation of *glpQ1* and *glpQ2*, that encode glycerophosphodiester phosphodiesterases, and are regulated by PhoP and also by glycerol and other carbon sources. PHO boxes have been identified in the promoter regions of *glpQ1* and *glpQ2* but the putative regulatory proteins recognizing glycerol or other carbon sources have not been studied yet (Santos-Beneit et al., 2009a). Increasing evidence indicates that coordination of phosphate and carbon source utilization, e.g., through the glycolysis have an important role in the metabolism of *Streptomyces* species. Recently, Ordóñez-Robles et al. (2017a) reported that in the tacrolimus producer *S. tsukubaensis* the use of glucose (but not maltose) as carbon source promotes drastically the expression of *phoR–phoP*, *pstS*, and other key genes of the Pho regulon. The findings that in *S. tsukubaensis* glucose stimulates significantly the genes involved in phosphate transport, and the similar effect of fructose in *S. lividans* indicates that an increase in the concentration of these hexoses in the culture medium favors the uptake of inorganic phosphate to balance the carbon and phosphate metabolism through the glycolysis, pentoses phosphate pathway, and TCA cycle. Reciprocally, inorganic phosphate affects many of the genes of the carbon metabolism and this regulation is mediated by PhoP (Rodríguez-García et al., 2007; Allenby et al., 2012), including enzymes involved in glycogen metabolism and gluconeogenesis (Thomas et al., 2012).

## Regulation of Nitrogen Metabolism in Actinobacteria: the GlnR Master Regulator

One of the most interesting examples of cross-talk between transcriptional regulators is the overlapping binding of PhoP and GlnR in the promoters of several nitrogen metabolism genes. *Streptomyces* and other Actinobacteria use a variety of nitrogen sources for growth. These include ammonium ions, nitrate and nitrite, and complex organic nitrogen sources including urea, amino acids, peptones, and several other nitrogen-containing compounds, including polyamines (Krysenko et al., 2017). In addition, some Actinobacteria, e.g., *Frankia* species are able to utilize atmospheric nitrogen for the production of organic nitrogen compounds in the cells (Nouioui et al., 2019). These different nitrogen sources are converted to glutamate and glutamine that serve as amino donors in transamination reactions to form other nitrogen containing compounds. In *Streptomyces* species there are two glutamine synthetases, encoded by the genes *glnA* and *glnII*, that convert glutamate to glutamine but the major glutamine synthetase activity is that of GlnA (Fink et al., 2002). Other glutamine synthetase-like enzyme, e.g., GlnA3, is known to be involved in polyamine utilization but has no glutamine synthetase activity (Krysenko et al., 2017). The ammonium ions are transported in the cells by the *amtB* transporter, which is clustered with genes *glnK* and *glnD*. On the other hand, nitrate and nitrite ions are reduced to ammonium by the nitrate reductases NasA (Amin et al., 2012), and nitrite reductase NirB1B2C/D (Tiffert et al., 2011). The urea is metabolized by a system encoded by the *ureABC* cluster.

All nitrogen metabolism genes respond to the nitrogen availability in the culture (Tiffert et al., 2008). Two regulatory genes, *glnR* and *glnRII*, control expression of several nitrogen metabolism genes at the transcriptional level (Fink et al., 2002). GlnR (267 amino acids) contains the domains defining a response regulator but is not linked to a cognate sensor kinase (Wray et al., 1991; Fink et al., 2002) and is classified as an orphan response regulator (Hutchings et al., 2004). It has been shown that GlnR in *Streptomyces* species is atypical in its lack of phosphorylation of the D^49^ residue (Hong et al., 2007); in PhoP this amino acid in its phosphorylated form activates the DBD but this activation does not seems to be required in GlnR. The protein region surrounding D^49^ is phosphorylated in serine and threonine residues and is essential for maintaining the biologically active homodimer conformation (Lin et al., 2014). Nucleotide sequences for GlnR binding upstream of many different nitrogen metabolism genes were shown to correspond to 22 nucleotides forming a direct tandem repeat of 11 nucleotides each (Tiffert et al., 2008; Sola-Landa et al., 2013). Under nitrogen limitation conditions GlnR induces expression of *glnA*, *glnII*, and the *amtB–glK–glD* cluster, but represses the cluster of urea utilization genes.

### Post-translational Phosphorylation or Acetylation of GlnR Affect Nitrogen Regulation in *S. coelicolor*

Mass spectrometry analysis of GlnR showed that this regulator is post-translationally phosphorylated at serine/threonine residues in six positions and acetylated at four lysine residues. The degree of phosphorylation correlates well with growth on nitrogen rich medium and is reduced, and even absent, in nitrogen limiting minimal medium. DNA binding studies using EMSA showed that the phosphorylated form of GlnR does not bind to its target sequences. Moreover, initial structural studies of GlnR indicated that the serine/threonine phosphorylated residues prevent formation of the active GlnR dimeric form (Amin et al., 2016). Overimposing the amino acid sequences of GlnR with the crystal structure of PhoP it was observed that the serine/threonine residues which are phosphorylated and the lysine residues which are acetylated in GlnR are conserved with respect to the same amino acids in PhoP. Regarding modification by acetylation, four lysine residues are acetylated in GlnR in nitrogen limiting medium but only one lysine is modified in nitrogen rich conditions. Therefore, it was proposed that the modifications in GlnR are similar to those reported in PhoP except phosphorylation at D^49^. However, the lysine acetylation does not appear to respond to the nitrogen availability in the medium but rather it responds to carbon sources (Amin et al., 2016), as described above for lysine acylation in PhoP in response to propionate or propanol.

### GlnR Regulates Secondary Metabolites Biosynthesis Through Pathway Specific Regulators

Detailed studies on nitrogen regulation mediated by GlnR were performed in the model actinobacteria *S. coelicolor* (Tiffert et al., 2011) and in *Streptomyces venezuelae* (Pullan et al., 2011). Other studies have been performed in *Sac. erythraea* (Yao et al., 2014), in *Amycolatopsis mediterranei* (Yu et al., 2007), and also in *S. hygroscopicus* var. *jinggangensis*, producer of validamycin (Qu et al., 2015). Transcriptional studies of a *S. coelicolor* mutant deleted “in frame” in *glnR*, confirmed that GlnR represses actinorhodin biosynthesis, and induces undecylprodigiosin formation. These observations were supported by DNA binding studies using purified GlnR. Noteworthy, it was found that GlnR binds directly to the promoter regions of genes for the pathway specific regulators ActII-orf4 and RedZ, respectively. He J.M. et al. (2016) identified a GlnR binding nucleotide sequence, 5-GTGAC-3, in the promoter regions of *actII-orf4* and *redZ* genes that has similarity to the nucleotide sequence of the A fragment in GlnR binding site described by Tiffert et al. (2008). Mutation of nucleotides in this putative GlnR binding site supported the conclusion that GlnR binds to the promoters of actinorhodin and undecylprodigiosin gene clusters in a sequence which is slightly different from those identified in primary nitrogen metabolism genes. It is possible that to bind these different sequences GlnR needs cooperation with either a sigma factor or other transcriptional regulators. In the case of genes for nitrate–nitrite assimilation, the recognition by GlnR is promoted by NnaR, which might act as a GlnR co-activator for *nirB1* expression (Amin et al., 2012).

In *S. avermitilis*, which produces the antihelminthic avermectin and the unrelated macrolide oligomycin, a mutant deleted in the *glnR* gene reduces drastically the production of avermectin but increased the biosynthesis of oligomycin (He J.M. et al., 2016). Transcriptional studies of the two regulatory genes involved in the control of avermectin, *aveR*, and oligomycin, *olmR*, indicate that GlnR in *S. avermitilis* binds to the promoter regions of these two regulatory genes. A very interesting aspect of this regulation in *S. avermitilis* is that the regulatory gene *aveR* contains also consensus binding sequences for PhoP (Yang et al., 2015; Martín et al., 2017), suggesting that there are regions upstream of the avermectin regulatory gene that integrate the response to different nutritional signals.

In summary, it may be concluded that GlnR is not only a master transcriptional regulator of nitrogen metabolism but it also coordinates primary metabolism and expression of secondary metabolites biosynthetic gene cluster through binding to the promoters of specific regulators of those gene clusters.

## Cross Talk of Phosphate and Nitrogen Regulation in *Streptomyces*

One of the most interesting recent development on interaction between master regulators is the cross-talk between GlnR and PhoP in the control of primary metabolism and also secondary metabolites biosynthesis in Actinobacteria.

**FIGURE 2:**
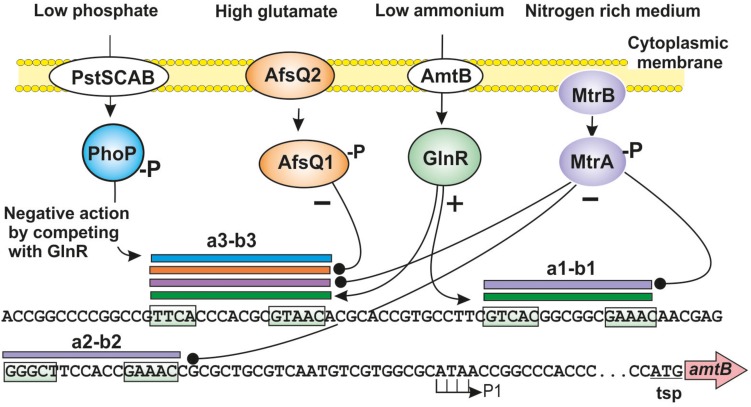
Signal integration sites upstream of *S. coelicolor amtB* gene. Interaction of the regulatory proteins GlnR (green sphere), PhoP (blue sphere), MtrA (purple sphere), and AfsQ1 (orange sphere) on the *amtB* promoter region. The binding sites of GlnR, PhoP, MtrA, and AfsQ1 on the a1-b1, a2-b2, and a3-b3 sites are indicated with green, blue, purple, and orange horizontal bars, respectively. The transcription start point (P1) and the translation start point (tsp) of *amtB* are indicated. Positive regulation is indicated by arrows and negative regulations by black spheres. The consensus GlnR binding sequences are boxed. See text for additional details.

PhoP and GlnR are related proteins of the OmpR response regulators family (Hutchings et al., 2004; Martín et al., 2012b). The expression of several genes involved in nitrogen metabolism is increased in a *S. coelicolor*Δ*phoP* mutant (Rodríguez-García et al., 2007). The control of PhoP over these genes is exerted in two ways. On the one hand, there is a binding of PhoP to the promoter of the *glnR* gene decreasing its expression when phosphate is limiting (Rodríguez-García et al., 2009; Allenby et al., 2012). In addition, PhoP binds directly to the promoters of at least six other genes related to nitrogen metabolism: *glnA*, *glnII*, the *amtB*–*glnK*–*glnD* operon, *ureA*, and some additional uncharacterized genes, interfering with the GlnR activation of these genes (Rodríguez-García et al., 2009; Sola-Landa et al., 2013). The interaction of PhoP with GlnR differs depending on the relative position of the sequences recognized by each of these proteins. In the *glnA* promoter, there is a direct competition between PhoP and GlnR because both regulators recognize overlapping sequences. In the *amtB* promoter, the PhoP and GlnR binding sequences are not fully overlapping, although they are very close and the binding of PhoP may alter the correct recognition of GlnR (Sola-Landa et al., 2013; Figure 2).

### The Nitrogen Regulator GlnR Controls Phosphate Transport by the PstS System in *A. mediterranei*

The interplay between GlnR and PhoP in *S. coelicolor* is well studied, but it is unknown if these conclusions are valid for other actinobacteria. A relevant example is the interaction of these regulators in *A. mediterranei*, a strain of industrial importance that produces rifamycin. Zhang et al. (2018) cloned the *pstSCAB* cluster of *A. mediterranei* and demonstrated its involvement in phosphate transport under phosphate limiting conditions. A mutant disrupted in the *pstS* gene showed a decreased production of rifamycin and, interestingly, revealed different morphology (swollen, near spherical cells rather than filamentous hyphae).

The *pstSCAB* operon was expressed as a transcriptional unit in *A. mediterranei* (Zhang et al., 2018) as reported also in *S. coelicolor* (Esteban et al., 2008) and confirmed by RNAseq studies (Martín-Martín et al., 2019). Nitrate has a well-known stimulatory effect on rifamycin biosynthesis by *A. mediterranei.* Transcriptional analysis revealed that formation and release of the PstS protein was induced by nitrate and regulated positively by GlnR. Expression of *pst*SCAB in a *glnR-*null mutant of *A. mediterranei* revealed a fourfold decrease of *pst*S transcription even in the presence of added nitrate; in other words, the nitrate stimulation of *pst*S expression is dependent upon GlnR. DNA binding studies with purified GlnR proved that this regulator binds to the upstream region of *pst*S, thus confirming that the high affinity phosphate transport system is under the control of GlnR in this actinobacteria. As indicated above, the *pstSCAB* cluster of many actinobacteria is strongly regulated by PhoP and, indeed, there is a PhoP binding sequence upstream of the *pstSCAB* genes in many bacteria. Therefore, the DNA sequences upstream of *pstSCAB* constitute an integrator site that combines signals of phosphate starvation and nitrogen availability (Zhang et al., 2018).

## Interplay of the Developmental Regulator MtrA With GlnR: Competition for the GlnR Binding Site

In 2017, it was observed that the response regulator MtrA (master transcriptional regulator A), member of the MtrAB TCS has an important role in nitrogen regulation (Som et al., 2017a, b; Zhang et al., 2017). The *mtrA* gene was initially discovered in *Mycobacterium tuberculosis*, where it has an important role in cell development and antigen secretion, and was also characterized in *C. glutamicum* (Möker et al., 2004). In *S. coelicolor* the MtrA protein is well conserved compared to the homologous proteins of *M. tuberculosis* and *C. glutamicum.* Initial studies indicated that in *S. coelicolor* MtrA has an important role in cell development since mutation of this regulator results in the lack of mycelium formation (*bald* phenotype) and also alters secondary metabolism (Som et al., 2017a, b). These studies revealed that MtrA binds a 17 nucleotides sequence consisting in two repeats of six nucleotides, separated by a five nucleotides non-conserved stretch, that surprisingly, overlap with the GlnR binding site in several nitrogen metabolism genes.

### Role of the Two Components System MtrAB in Nitrogen Metabolism in *S. coelicolor*

Recently, it was experimentally demonstrated that MtrA binds to one of the three putative GlnR binding sequences upstream of the *amtB–glnK–glnD* cluster (Zhu et al., 2019). Previously, three putative GlnR binding sites (named a1-b1, a2-b2, and a3-b3), each consisting in two binding repeats had been identified in the *amtB* promoter region (Wang et al., 2012; Li et al., 2018). More recently, Zhu et al. (2019) showed that the developmental regulator MtrA recognizes the a3-b3 binding sequence which also binds GlnR efficiently. This was confirmed by EMSA DNA binding assays using the a3-b3 sequence as probe (Figure 2). MtrA was also able to bind the a1-b1 and a2-b2 sites, although GlnR did not recognizes the a2-b2 site indicating that minor differences in nucleotides in these binding sequences determine the affinity of binding for MtrA versus GlnR. Additionally, binding of MtrA to GlnR boxes upstream of other nitrogen metabolism genes such as *glnA*, *glnII*, *nirB1B2C/D*, and *ureA* was also found. In all cases MtrA was bound to the GlnR recognition sites although the affinity of binding changes depending on the nucleotide sequences of the different GlnR boxes. It is worth to note that the a3-b3 site was also recognized by PhoP (Figure 2; Wang et al., 2012; Sola-Landa et al., 2013).

Similarly, competition experiments and EMSA assays on the *nirB1B2C/D* promoter using different amounts of MtrA and GlnR showed that the intensity of the DNA–protein complexes varies depending on the relative amount of MtrA to GlnR. In other words, both proteins compete for the same nucleotide sequence, demonstrating that there is an overlapping binding of the two regulators to the upstream sequence of the *nirB1B2C/D* cluster.

*In vivo* binding of MtrA to GlnR recognition sequences in different nitrogen metabolism genes was shown by ChIP-qPCR analysis using anti-Flag antibodies and a *S. coelicolor* Δ*mtrA* mutant transformed with the Mtr-Flag fused gene (Zhu et al., 2019). Comparative transcriptional analysis of gene expression in the parental strain and the *mtrA-*deleted mutant showed that several nitrogen metabolism genes including the *amtB*–*glnk*–*glnD* cluster, *glnII*, *nasA*, and *nirB1*–*nirB2*–*nirC/D* were highly overexpressed in the *mtrA*-deleted mutant under nitrogen rich medium whereas the urea utilization cluster was not significantly upregulated under these conditions.

These results clearly indicate that under nitrogen rich conditions MtrA represses nitrogen metabolism genes, i.e., this is a regulation opposed to that of GlnR in minimal medium. Interestingly, comparative transcriptional experiments were also performed under nitrogen limiting conditions using the *mtrA-* or the *glnR*-deleted mutant, compared to the parental strain (Zhu et al., 2019); the results clearly indicated that expression of several nitrogen metabolism genes respond to the GlnR concentration in the cells but not to the MtrA levels. In other words under nitrogen limiting conditions the positive regulatory effect of GlnR dominates over the MtrA repression.

Noteworthy, although GlnR does not regulates its own expression, binding of the purified MtrA protein to a sequence upstream of *glnR* was found. Indeed, there was no binding of purified GlnR to the upstream sequence of the *glnR* gene itself, although it is recognized by MtrA. These results are difficult to explain if it is assumed that both GlnR and MtrA recognized the same binding sequences. Probably other proteins interact with MtrA and modify its affinity compared to that of GlnR.

Similarly, NtrA of *S. lividans* regulates expression of several nitrogen metabolism genes under nitrogen rich conditions, as occurs in *S. coelicolor* (Zhu et al., 2019). Furthermore, the *ntrA* gene has been characterized in *M. tuberculosis* and *C. glutamicum*. Initial experiments using NtrA purified protein from these actinobacteria indicate that potentially MtrA is also able to regulate nitrogen metabolism genes both in *Mycobacterium* and in *Corynebacterium*. It is likely that a similar regulatory mechanism occurs in other actinobacteria. In summary, the wide distribution of genes encoding GlnR and MtrA in several actinobacteria suggests that these two master regulators play a very important role in nitrogen metabolism.

### Interplay of Nitrogen and Carbon Regulatory Mechanisms Mediated by GlnR

Early studies on GlnR-mediated regulation of metabolism in *Streptomyces* suggested that this regulator is also involved in the control of carbon sources assimilation (Tiffert et al., 2008; Pullan et al., 2011).

**FIGURE 3:**
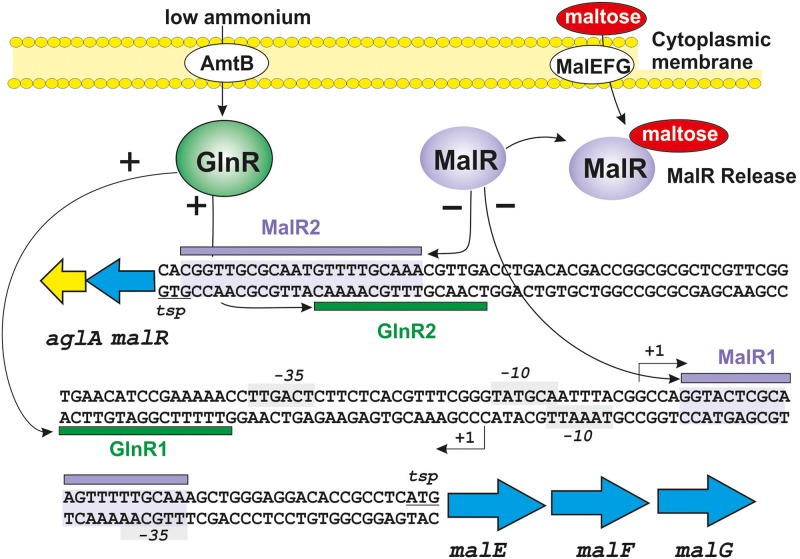
Interaction between the repressor protein MalR and the activator protein GlnR on the *mal* operon of *Sac. erythraea*. The intergenic *aglAmalR-malEFG* region is shown with the MalR binding sites shadowed in purple. The binding sites for MalR and GlnR are indicated with purple and green horizontal bars, respectively. The -10 and -35 sites for both operons are shown in gray boxes. The +1 transcription sites and the translation starting points for both operons are indicated. Positive regulation is indicated by arrows and a (+) sign; negative regulation is indicated by arrows and a (−) sign. Maltose (red ellipse) induces the *malEFG* operon by binding to the MalR repressor and releasing it from the DNA. See text for additional details.

An interesting recent development is the finding that GlnR interacts with metabolism of many carbon sources at the transport level (Liao et al., 2015). The actinobacteria use a large variety of carbon sources (Bertram et al., 2004; Titgemeyer et al., 2007). Some of them, e.g., glucose or glycerol, are preferred carbon sources but the utilization of many other hexoses, oligosaccharides, and polysaccharides is non-constitutive and is only induced when required. In *Streptomyces* species, glucose is transported by a well-known permease system (van Wezel et al., 2005). In contrast 87% of the total carbohydrate carbon sources are transported by ATP binding cassettes (ABC) transporter systems (van Wezel et al., 1997; Davidson et al., 2008). Studies on the control by GlnR of several ABC sugar transporters have been done in *Sac. erythraea* and in *S. coelicolor* (Liao et al., 2015). These authors identified a sequence similar to the GlnR-binding site in the upstream region of the *malEFG* operon of *Sac. erythraea*, that encodes an ABC transport system for maltose and maltodextrines (Figure 3). Immediately upstream of the *malEFG* operon in the complementary strand and in the opposite orientation are two genes *malR* and *aglA*, encoding the MalR repressor protein and an α-glucosidase involved in the cleavage of maltose and maltodextrins to produce glucose. Both in *Sac. erythraea* and in *S. coelicolor* two MalR binding sites were detected and confirmed in DNA binding assays in the intergenic region *malEFG-malR*, one of which overlap with the GlnR binding sequence (Liao et al., 2015; Figure 3). Mutants of *Sac. erythraea* or *S. coelicolor* deleted in the *glnR* gene showed impaired growth in media containing maltose as only carbon source but not in media supplemented with glucose. Transcriptional studies revealed that expression of the *mal* operon was dependent on the GlnR regulator, i.e., there is no expression of this operon in the *glnR*-deleted mutant. Binding of purified GlnR to the upstream region of the *mal* operon was shown by DNA binding assays. Transcription of the *mal* operon increases in response to nitrogen starvation, indicating that the nitrogen availability controls expression of the *mal* transport system (Figure 3).

In *S. coelicolor* 12 out of 37 putative ABC sugar transporters were found to contain GlnR binding sites. The same situation was found in *A. mediterranei*, *Mycobacterium smegmatis*, and three additional *Streptomyces: S. griseus*, *S. venezuelae*, and *S. avermitilis* (Liao et al., 2015). Similarly, GlnR binding sites were found in 8 of the 19 putative ABC-type sugar transporters in *Sac. erythraea*. It seems that in all these cases expression of the sugar binding subunits of the respective ABC transporters was dependent on the GlnR regulator.

Altogether these findings indicate that there is a cross-talk between the limitation of nitrogen sources and the transport of several sugars by ABC systems. This interplay between the nitrogen regulation and sugar transport system protects the cell against imbalances in nitrogen and carbon abundance by controlling the utilization of alternative sugar sources. When nitrogen is limiting the GlnR levels increase, and therefore favor the utilization of alternative nitrogen (Tiffert et al., 2011) and carbon sources (Liao et al., 2015).

## Possible Interaction Between the cAmp Receptor Protein and the GlnR Nitrogen Regulator

Glucose is the preferred carbon source by *Streptomyces* and many other bacteria, and there is a regulation by glucose of alternative non-preferred carbon sources utilization. This regulation in Enterobacteria is mediated by the cAMP receptor protein (CRP) and is dependent on the cAMP levels in the cells. Several studies have reported that in *S. coelicolor*, the carbon catabolite regulation of alternative carbon sources by glucose is not mediated by a CRP-homologous protein. Instead, carbon catabolite regulation is mediated by the glycolytic enzyme glucose kinase (Ikeda et al., 1984; Angell et al., 1994; van Wezel et al., 2007; Romero-Rodríguez et al., 2016a, b). However, in *Sac. erythraea* there is a CRP-like protein that seems to be involved in carbon catabolite control of utilization of alternative carbon sources. This regulation involves binding of the CRP-like protein to promoter regions of several ABC-type sugar transporters. Bioinformatic studies revealed that there are putative CRP binding sequences in the upstream regions of several ABC sugar transporters in *Sac. erythraea* and possibly also in other actinobacteria (Liao et al., 2015). This finding adds a new level of regulation by interaction of CRP with promoters of ABC sugar transporters, such as *malEFG*. Indeed, Liao et al. (2015) proposed that CRP binds to the same region recognized by GlnR in the intergenic region between *malEFG* and *malR-aglA* (Figure 3). However, the putative interaction at this level needs further experimental confirmation. In summary, the possible interaction between the CRP protein, the GlnR and the MalR regulator in the *mal* operon for maltose/maltodextrins metabolism, is another provocative example of interaction of several regulatory proteins on the promoter region of key sugar transporters and therefore constitutes an elaborate mechanism of control by interacting transcriptional regulators.

## Interplay Between the Two Components System A*fs*Q1/Q2 and the Nitrogen Regulator GlnR

The two components system AfsQ1–AfsQ2 (of which *afsQ1* encodes the response regulator) was initially found as a result of the observation that it increases the production of actinorhodin and undecylprodigiosin in *S. coelicolor* (Shu et al., 2009). In the opposite orientation of *afsQ1–AfsQ2* is located the *sigQ* gene, encoding a putative sigma factor involved in antibiotic biosynthesis, that is regulated by AfsQ1 (Wang et al., 2013). The observed regulation takes place only in minimal medium supplemented with 75 mM glutamate. Transcriptional studies using cell grown under these glutamate rich conditions revealed that the expression of *actII-orf4*, *cdaR*, and *redZ* genes was significantly reduced in the *afsQ1-Q2* mutant. EMSA studies with purified AfsQ1 revealed that, indeed, this response regulator was able to bind to the *actII-orf4*, *cdaR*, and *redZ* promoters but not to the *redD* promoter region. The AfsQ1 regulator enhances the expression of *sigQ* but not that of the *afsQ1–afsQ2* operon itself. In other words, there is a bidirectional transcription region that expressed *afsQ1-Q2* in one orientation and *sigQ* in the other and AfsQ1 does not regulates directly its own expression. The AfsQ1 binding sequence was first identified in the promoters of *sigQ* and *redZ* and later also in *actII-orf4* and *cdaR*. In all cases, except in *actII-orf4*, a 16 nucleotides sequence was protected by AfsQ1. This sequence, GTnAC-n6-GTnAC, consists of two repeated GTnAC nucleotides sequences separated by an intervening non-conserved six nucleotides stretch. Surprisingly, the sequence recognized by AfsQ1 in *actII-orf4* does not adjust to the consensus five nucleotides repeated sequence indicating that there are factors, still unknown, that affect the specificity of the binding sequences recognized by AfsQ1 (Wang et al., 2013). These authors using a matrix based on the conserved 16 nucleotides binding sequence were able to recognize 17 sites that correspond to: (1) the upstream regions of nitrogen metabolism genes, such as *glnA* and *nirB1*; (2) the *pstS* gene involved in high affinity phosphate transport; and (3) several bidirectional promoters related to cell differentiation including *whiD* and *bldM* (Wang et al., 2013).

The putative AfsQ1-binding sequence contains the same five nucleotide core repeats that form part of the 16 nucleotides consensus sequence recognized by GlnR. EMSA studies with purified AfsQ1 revealed that this regulator binds to the promoter regions of several nitrogen metabolism genes but not to the promoter of *glnR* or *glnII* indicating that there are differences in the affinity of distinct regulatory proteins for nitrogen metabolism genes. Transcriptional studies using a mutant deleted in *afsQ1*–*afsQ2* showed that AfsQ1 acts as a transcriptional repressor of several nitrogen assimilation genes under conditions of high glutamate concentration. Competition DNA binding experiments using both purified AfsQ1 and GlnR proteins lead to the conclusion that both regulators compete for the same nucleotide sequence in the upstream region of several nitrogen assimilation genes; in other words, AfsQ1 represses the expression of genes encoding enzymes for assimilation of alternative nitrogen sources in conditions of glutamate excess by competing with GlnR that would activate these promoters under nitrogen limitation conditions.

An important observation is the finding that AfsQ1 regulates expression of the *pstSCAB* cluster that is also strongly regulated by PhoP and AfsR (Santos-Beneit et al., 2009b; Martín et al., 2012a). Indeed, PhoP also regulates expression of several nitrogen assimilation genes as described above in this article. Taken together all this information suggests that these five master regulators PhoP, AfsR, AfsQ1, GlnR, and MtrA, interact in the fine tuning of regulation of key genes in carbon, nitrogen, and phosphate metabolism. This interaction protects the cell against unbalance metabolism due to lack or excess of some key nutrients, such as ammonium or glutamate (Table 1 and Figure 2). Not all five regulators bind to the same integrative node; two, three, or more transcriptional factors may recognize certain sequences whereas combinations of other factors regulate expression of genes at alternative nodes.

## Overlapping Transcriptional Factors Regulate Expression of AtrA

Other interesting example of interplay between transcriptional factors is the cross regulation between PhoP and the AdpA regulator, that in turn responds to the γ-butyrolactone signaling mechanism. In the quorum sensing system the γ-butyrolactones are recognized by specific butyrolactone receptor proteins (Brps) (Horinouchi, 2002; Ohnishi et al., 2005) that when combined with its own cognate butyrolatone derepressed the binding of the Brps to the pleiotropic regulator AdpA (Takano, 2006). This occurs in different *Streptomyces* species (Higo et al., 2012; Salehi-Najafabadi et al., 2014) and has been studied with detail in *Streptomyces roseosporus*. The butyrolactone triggered signaling cascade in *S. roseosporus* is known to proceeded through the TetR-type regulator AtrA, that controls expression of the biosynthesis of the lipopeptide antibiotic daptomycin (Mao et al., 2015). Expression of AtrA is controlled by the pleiotropic regulator AdpA. Recently, Zheng et al. (2019) observed that expression of AtrA is regulated in addition by PhoP. Binding of these two regulators overlaps in the upstream region of the *atrA* gene as confirmed by DNA binding studies. Moreover, in turn PhoP indirectly controls expression of the *adpA* gene (Allenby et al., 2012). Therefore, PhoP controls AtrA at two different levels in a cumulative form. In conclusion, this is other excellent example of overlapping regulation exerted by two different master regulators, PhoP and AdpA, on the expression of the *atrA* gene. Since AdpA is regulated by the γ-butyrolactone quorum sensing system the overall mechanism connects the regulation by small ligands (Xu et al., 2010; Martín et al., 2019) with the regulation exerted by the master regulators of phosphate metabolism.

## Binding of Interacting Transcriptional Factors: the Concept of Signal Integrative DNA Regions

Increasing evidence indicates that there are numerous examples of overlapping sequences that integrate nutritional and environmental stress signals in *Streptomyces* species (Romero-Rodríguez et al., 2018). As described above six or even more transcriptional factors may bind to a specific promoter region. Therefore, the concept of a metabolic safety net is of interest to understand how the interaction of these stress signals, through integrator DNA sites, protect the metabolism of the cells against nutritional and environmental damage by achieving a balance of the key metabolic pathways.

The evidence that different master transcriptional factors interact on overlapping DNA sequences has been supported by studies on ChIP-on-chip analysis. This technique that uses antibodies against one of the binding proteins immunoprecipitates the DNA region bound by the specific factor targeted by the antibody and also results in coprecipitation of other interacting proteins (Allenby et al., 2012). This study indicated that there are many more binding sites for PhoP or other transcriptional factors, e.g., DasR (Świa̧tek-Połatyńska et al., 2015), than those expected from previous transcriptomic studies. The authors propose that interaction of PhoP with other transcriptional factors increase the affinity of PhoP for some poorly conserved PHO boxes. For example, Allenby et al. (2012) observed a massive binding of PhoP to the *cpk* gene cluster corresponding to internal sequences of *cpkB* and *cpkC* genes involved in coelimycin P1 biosynthesis (Gomez-Escribano et al., 2012). It is possible that this intense PhoP binding may be due to some interaction with some other factors that control those genes in the *cpk* gene cluster.

Moreover, these master regulators in turn control several other transcriptional factors at lower levels, that regulates differentiation, osmotic stress responses, oxygen metabolism, and secondary metabolite biosynthesis.

## Additional Intertwining Layers of Regulation Reinforce the Safety Net

**FIGURE 4:**
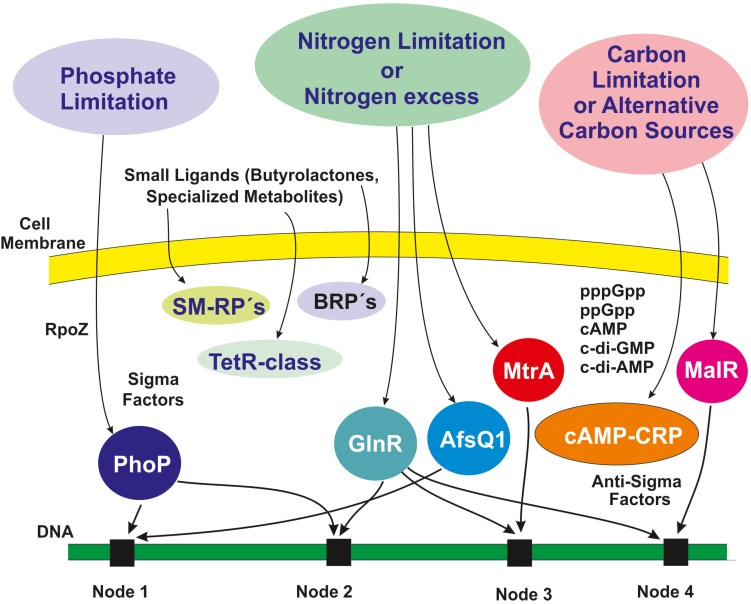
Schematic representation of overlapping layers of regulation that reinforce the safety net. The outer circles represent nutritional stresses that elicit regulatory responses (detailed in Figures 1–3). Additional regulatory elements including sigma factors, highly phosphorylated and cyclic nucleotides are shown inside the cell. Receptors proteins (SM-RPs, BRPs) and the TetR-class transcriptional regulators are receptors of signal molecules that are secreted and re-enter the cell. Four integration nodes are highlighted in black boxes in the DNA. The different regulatory sigma and anti-sigma factors and the ligand receptor proteins are connected to distinct nodes (not shown; see text). BRPs, γ-butyrolactone receptor proteins; SM-RPs, specialized metabolites receptor proteins; CRP, cAMP receptor protein.

In this article we have focused on interactions of transcriptional factors that control expression of different gene clusters in *Streptomyces*. These interactions constitute the backbone of the safety net but there are many other regulatory factors at distinct levels that provide different layers of control (Figure 4). These include sigma factors and anti-sigma factors (Paget, 2015), guanosine tetraphosphate, guanosine pentaphosphate, and novel cyclic guanine and adenine nucleotides, all of which act the transcriptional level, and ancillary subunits of the RNA polymerase. In this article we are concentrating on the interactions at the transcriptional level. Life of Actinobacteria is subject to frequent changes in nutritional and environmental conditions and this requires adaptation to the stress produced by those changes. To adjust to those changing conditions Actinobacteria have developed a variety of alternative sigma factors that recognize and activate adequate promoters in response to the novel conditions. Housekeeping genes that are strictly required for growth are transcribed by RNA polymerase that contain sigma factors of the Sig70 family, namely the HrdB factor; however, there are several paralogous genes similar to *hrdB* (e.g., *hrdA*, *hrdC*, and *hrdD*), whose function is still partially unclear. Sequencing of *Streptomyces* genomes has revealed that these actinobacteria contain many alternative sigma factors, 69 (66 in the chromosome and 3 in plasmids) in *S. coelicolor* (Bentley et al., 2002) and 62 in *S. lividans* (Rückert et al., 2015; Rebets et al., 2018), whereas *Escherichia coli* contains only 7 sigma factors and *B. subtilis* has 18. Alternative sigma factors play an important role on expression of genes involved in many aspects related to growth, differentiation, stress response, and secondary metabolites biosynthesis (Sun et al., 2017). Therefore, the interactions of these alternative sigma factors with promoter regions that also are regulated by the above described transcriptional factors provide an intertwining mechanism of control of gene expression. Several sigma factors are antagonized by anti-sigma proteins (19 in the *S. lividans* genome). The result is that the interaction of the cognate sigma factor with the RNA polymerase is blocked by binding of the corresponding anti-sigma factor. This adds an additional level of control on the interaction of transcriptional factors and sigma factors in the safety network. An additional protein that interacts with the RNA polymerase core enzyme is the ancillary protein RpoZ that in *S. coelicolor* is controlled by the phosphate regulator PhoP; mutant in *rpoZ* are impaired in cell differentiation and secondary metabolites biosynthesis (Santos-Beneit et al., 2011a).

Another group of molecules that interact with the RNA polymerase are the guanosine hyperphosphorylated molecules, namely guanosine tetra-phosphate (ppGpp) and guanosine pentaphosphate (pppGpp) These compounds initially characterized in *E. coli* have been found in many other bacteria including actinobacteria. These molecules are formed in response to carbon and nitrogen limitation, particularly in amino acid starvation caused be the absence of an essential amino acid (Dalebroux and Swanson, 2012) and constitutes the so called stringent response that limits transcription of several genes involved in vegetative growth (e.g., genes involved in ribosomal protein synthesis) and stimulates expression of stress response genes. The highly phosphorylated guanosine nucleotides are synthesized by a ppGpp synthetase encoded by the *relA* gene. In Enterobacteria the ppGpp is degrade by a ppGpp phosphohydrolase whereas in *Streptomyces* the *relA* gene appears to have both biosynthetic and degradative activities, being named *relA*/*spoT* (Martínez-Costa et al., 1998). Based on the information derived from *S. coelicolor relA* gene it was proposed that accumulation of ppGpp in response to nitrogen limitation increases biosynthesis of secondary metabolites (Bibb, 2005). Indeed, this increase is mediated by the extracytoplasmic factor SigT, thus providing an interesting example of interaction between sigma factors and stringent response (Feng et al., 2011). Mutants deleted in the *relA* gene showed greatly reduced transcription of the *actII-orf4* and *redD* genes, which are required for expression of the *act* and *red* gene clusters (Chakraburtty and Bibb, 1997; Hesketh et al., 2001). Global transcriptomic studies comparing the S. *coelicolor* parental strain and the *relA*-null mutant showed that *relA* controls expression of 598 genes most of them involved in growth and differentiation and some of them affecting secondary metabolite biosynthesis (Hesketh et al., 2007). Among the impacted genes was the major vegetative sigma factor HrdB that is repressed following ppGpp accumulation. The biosynthesis of alternative sigma factors was also affected in the *relA*-deleted mutant (Hesketh et al., 2001). However, more recent information indicates that biosynthesis of ppGpp is not strictly required for triggering biosynthesis of secondary metabolites, even in some *Streptomyces* species such as *Streptomyces clavuligerus* deletion of the *relA*-gene resulted in a 2–2.5-fold overproduction of cephamycin C and clavulanic acid, indicating that (p)ppGpp may influence positively or negatively antibiotic biosynthesis (Gomez-Escribano et al., 2008). The highly phosphorylated guanine nucleotides form part of a family of second messengers that have been paid more attention recently; these include in addition to ppGpp and pppGpp, cyclic adenosine 3′5′-monophosphate (cAMP), cyclic guanosine 3′5′-monophosphate (cGMP), and purine cyclic dimers as bis (3′,5′)-cyclic di guanosine monophosphate (c-di-GMP) or cyclic dimer adenosine monophosphate (c-di-AMP) (Latoscha et al., 2019). There is still little information on these second messenger nucleotides in *Streptomyces* species. A putative adenylate cyclase encoded by the *cyaA* gene was found in *S. coelicolor* (Danchin et al., 1993). Bioinformatic analysis reveals that the *S. coelicolor* adenylate cyclase encoded by *cyaA* has the same domains as the adenylate cyclase of *M. tuberculosis*. This mycobacterial protein has been crystalized and has been proposed to have a role on acid pH recognition (Tews et al., 2005). The *S. coelicolor cyaA*-null mutant is unable to neutralize the acidic pH produced during growth of the culture and is bald. The aerial mycelium formation was restored when exogenous cAMP was added to the mutant culture (Süsstrunk et al., 1998). Interestingly, a CRP protein has been identified in *S. coelicolor* and found to bind cAMP *in vitro* (Derouaux et al., 2004). However, cAMP does not seem to play a role similar to that of carbon catabolite regulation by glucose of alternative carbon sources. Noteworthy, immunoprecipitation studies have shown that CRP co-precipitates with the upstream region of *actII-orf4* and *redD* (Gao et al., 2012). cAMP has a CRP-dependent inducing effect on expression of these regulatory genes. These finding are consistent with the observation that CRP mutants are impaired in the biosynthesis of actinorhodin and undecylprodigiosin. In conclusion, it seems that the cAMP–CRP complex interacts with the upstream region of *actII-orf4* and *red* and, therefore, regulates expression of the corresponding gene clusters. The possible mechanism, if any, of cAMP–CRP on carbon catabolite regulation in *Streptomyces* needs to be further studied (see the section “Possible Interaction Between the cAMP Receptor Protein and the GlnR Nitrogen Regulator”).

Another modified nucleotide, c-di-GMP, has been studied recently in *S. venezuelae* (Al-Bassam et al., 2018). This nucleotide is synthesized by a dimeric guanylate cyclase using two GMP molecules as substrate. The c-di-GMP level is maintained by degradation catalyzed by a phosphodiesterase to form a linear GMP di-nucleotide. Similar enzymes exist in *S. coelicolor*. Disruption of genes encoding these c-di-GMP biosynthetic and degradative enzymes impaired actinorhodin and undecylprodigiosin formation but the mechanism involved in this effect is unknown. Cell differentiation and spore pigmentation were also affected in the c-di-GMP-null mutant (Tran et al., 2011; Hull et al., 2012). The last of this group of modified nucleotides is bis (3′,5′)-cyclic di adenosine monophosphate (c-di-AMP) that is formed by an adenylate cyclase from two ATP molecules. The genes forming c-di-AMP have been studied in *B. subtilis* and they are also present in actinobacteria including *Streptomyces* species (Witte et al., 2008). The intracellular balance of c-di-AMP is maintained by at least two different mechanisms, one of them involves secretion by a multidrug efflux pump and the second implicates hydrolysis by a phosphodiesterase. Interestingly, the gene encoding this enzyme, that occurs in other bacteria, is not present in *Streptomyces* genomes. The intracellular c-di-AMP levels affect many aspects of cell metabolism, including cell wall preservation and DNA integrity maintenance, but the major aspect regulated by this nucleotide is the ion homeostasis of the cell, particularly osmotic stress balance (Commichau et al., 2018). The interactions between the different second messenger nucleotides and phosphate control is still obscure. It is well known that synthesis of high levels of hyperphosphorylated guanine nucleotides results in a decrease of GTP pool in the cell and vice versa the level of GTP increases in the guanosine tetraphosphate null mutants. The cellular signals that regulate the levels of these modified nucleotides and their relationship with the global phosphate control need to be further investigated.

A further layer of interactions in the protection net is exerted by small ligands that bind to receptor proteins and caused distinct regulatory effects. These ligands are small organic molecules different from normal nutrients that are produced by bacteria and fungi in complex habitats and exert effect in the same species or between different species (Keller and Surette, 2006; Shank and Kolter, 2009; Straight and Kolter, 2009). Several of these ligands are themselves specialized metabolites that are secreted in small amount but exert potent communication effects on competing microorganisms. One group of these ligands are the so-called quorum sensing ligands (Bassler and Losick, 2006). One of the typical classes of quorum sensing molecules are the γ-butyrolactones that are well known in *Streptomyces* and related actinobacteria (Takano, 2006; Nodwell, 2014). The distinct γ-butyrolactones that differ in the modifications of their short fatty acid chains are recognized by γ-butyrolactone receptor proteins. Another important group of receptor proteins, that were initially designated pseudo-γ-butyrolactone receptor proteins (Xu et al., 2010), and have been renamed specialized metabolites receptor proteins (Martín and Liras, 2019), do not recognize γ-butyrolactones but interact with different biosynthetic intermediates and natural products that act as signals in the bacterial intercommunication. In addition to the specialized metabolite receptor proteins other well-known molecules recognize small ligands; this is the case of the TetR-class of transcriptional regulators. The TetR cluster regulators have a ligand binding site in the carboxyterminal region that interact with diverse signal molecules (Cuthbertson and Nodwell, 2013). In conclusion, the number of reported examples of interactions between ligands and transcriptional factors is increasing and the overall picture is that these additional layers of regulation reinforce the safety net with a more intricate connections between the nods. In addition, translational and post-translational regulatory mechanisms also reinforce the safety net.

### Related Protection Nets in Other Bacteria

This article focuses on the protection net in *Streptomyces* species and related actinobacteria but equivalent protection nets exist in many other bacteria and in lower eukaryotes. However, the molecular mechanisms that protect the cells in response to nutrient starvation and environmental stresses are rather different from those observed in *Streptomyces*. For example, in *E. coli* there are only seven alternative sigma factors. The mechanism of control of the Pho regulon in *E. coli* is somehow different from that observed in *Streptomyces* species (Hsieh and Wanner, 2010; Antiqueira et al., 2012). In contrast to the strict dependence on the sensor histidine kinase PhoR in *S. coelicolor* (Fernández-Martínez et al., 2012), in *E. coli* the phosphorylation of the response regulator PhoB occurs also by acetyl-phosphate. Moreover, the phosphorylation of the PhoB regulator is also performed by histidine kinases of other two-components systems, as is the case of CreC histidine kinase (Wanner, 1993; Hsieh and Wanner, 2010). Interplay of regulatory systems has been also studied in *B. subtilis* and again there are differences with respect to the *Streptomyces* network; for example, the phosphate starvation response is mediated by a phosphorelay mechanism that involves at least three different steps in a cascade through the PhoR–PhoP TCS, the Spo0 system, and the ResD/ResP system (Hulett, 1996). In conclusion, it is clear that different bacteria have adapted their control mechanisms to the habitat in which they survive and the network of response mechanisms is adapted to those specific niches.

## Conclusion

All available evidence indicates that there are signal integration sites in the genome of *Streptomyces* that are recognized by interacting transcriptional factors.

These nucleotide sequences upstream of key genes constitute integrative nodes that serve to compensate the unbalance produced by the lack of a key nutrient or for its excess that would impair the cell metabolism. Probably there are several hundreds of these integrator sites; however, it remains to be confirmed that some of these sites serve to integrate signals for the major transcriptional factors such a PhoP, GlnR, MtrA, and so on, whereas other sites probably integrate two or a few signals as appears to be the case of the *atrA* promoter region that is bound by PhoP and AdpA. It will be of utmost interest to establish the hierarchy of the integration sites to get a clear idea of the role of these sites on regulation of overall metabolism, particularly to advance our knowledge of the system biology of these actinobacteria.

Finally, an interesting conclusion is that mutations that initially may be designed to change a specific regulatory phenomenon that affects a single cluster may, however, alter the expression of many different clusters through the interplay of these major transcriptional factors. An intriguing question is whether the signal integration sites in the DNA correspond to open “unprotected DNA region” at difference of other DNA regions that may be protected by histone-like proteins.

## Author Contributions

All authors listed have made a substantial, direct and intellectual contribution to the work, and approved it for publication.

## Conflict of Interest

The authors declare that the research was conducted in the absence of any commercial or financial relationships that could be construed as a potential conflict of interest.
